# Crystal structure of 1-(2,4-dihy­droxy-6-methyl­phen­yl)ethanone

**DOI:** 10.1107/S2056989015013468

**Published:** 2015-07-29

**Authors:** Samran Prabpai, Palangpon Kongsaeree

**Affiliations:** a Department of Chemistry, and Center for Excellence in Protein Structure and Function, Faculty of Science, Mahidol University, Bangkok 10400, Thailand

**Keywords:** crystal structure, 1-(2,4-dihy­droxy-6-methyl­phen­yl)ethanone, bioactive secondary metabolite, hydrogen bonding, π–π stacking

## Abstract

The title compound, C_9_H_10_O_3_, is a bioactive secondary metabolite, isolated from the endophytic fungus *Nodulisporium* sp. The compound exhibits an intra­molecular O—H⋯O hydrogen bond between the phenolic H atom and the carbonyl O atom of the adjacent acetyl group. In the crystal, mol­ecules are linked by hydrogen bonds involving the 4-phenolic H atom and a symmetry-related carbonyl O atom of a neighboring mol­ecule, resulting in extended supra­molecular chains along the *a*-axis direction. Aromatic π–π stacking inter­actions between the nearly parallel benzene rings of adjacent chains [centroid–centroid distance = 3.7478 (8) Å] further stabilize the three-dimensional supra­molecular framework.

## Related literature   

For biological activities of aceto­phenone derivatives, see: Das & Khosla (2009 ▸); Suzuki *et al.* (2006 ▸); Tabuchi *et al.* (2014 ▸). For related structures, see: Azeezaa *et al.* (2009 ▸); Chakkaravarthi *et al.* (2007 ▸); Hill *et al.* (2012 ▸).
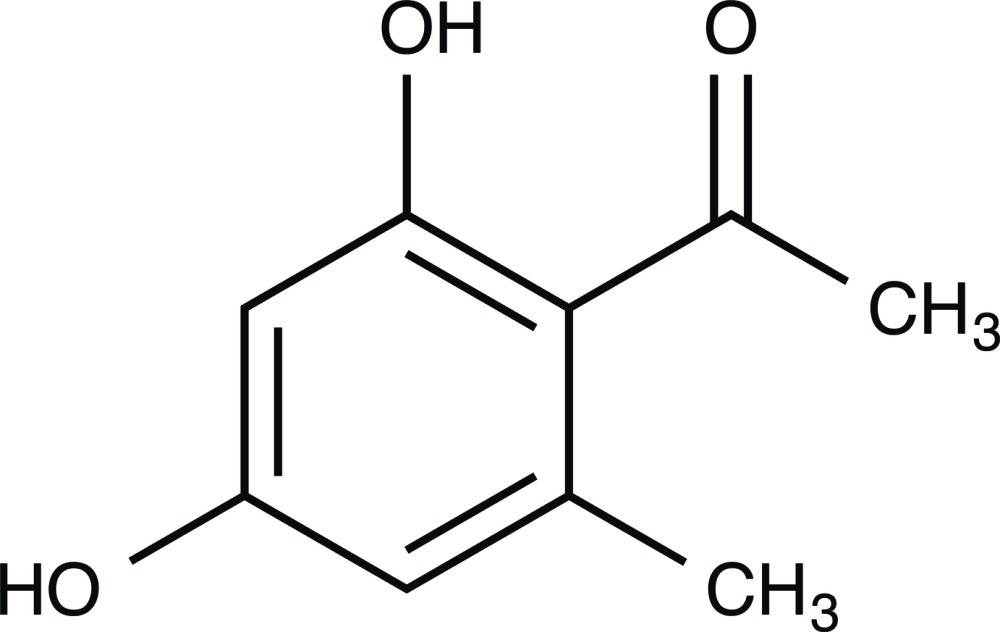


## Experimental   

### Crystal data  

C_9_H_10_O_3_
*M* *_r_* = 166.17
Monoclinic, 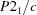

*a* = 7.3570 (3) Å
*b* = 15.001 (1) Å
*c* = 7.3180 (5) Å
β = 91.017 (4)°
*V* = 807.50 (8) Å^3^
*Z* = 4
Mo *K*α radiation
μ = 0.10 mm^−1^
*T* = 298 K
0.25 × 0.25 × 0.25 mm

### Data collection  

Nonius KappaCCD diffractometer
3260 measured reflections
1828 independent reflections
1319 reflections with *I* > 2σ(*I*)
*R*_int_ = 0.028

### Refinement  

*R*[*F* ^2^ > 2σ(*F* ^2^)] = 0.053
*wR*(*F* ^2^) = 0.150
*S* = 1.03
1828 reflections
114 parameters
H-atom parameters constrained
Δρ_max_ = 0.22 e Å^−3^
Δρ_min_ = −0.17 e Å^−3^

### 

Data collection: *COLLECT* (Nonius, 2000 ▸); cell refinement: *SCALEPACK* (Otwinowski & Minor, 1997 ▸); data reduction: *DENZO* (Otwinowski & Minor, 1997 ▸) and *SCALEPACK*; program(s) used to solve structure: *SIR97* (Altomare *et al.*, 1999 ▸); program(s) used to refine structure: *SHELXL2013* (Sheldrick, 2015 ▸); molecular graphics: *OLEX2* (Dolomanov *et al.*, 2009 ▸) and *Mercury* (Macrae *et al.*, 2008 ▸); software used to prepare material for publication: *publCIF* (Westrip, 2010 ▸).

## Supplementary Material

Crystal structure: contains datablock(s) I, New_Global_Publ_Block. DOI: 10.1107/S2056989015013468/xu5859sup1.cif

Structure factors: contains datablock(s) I. DOI: 10.1107/S2056989015013468/xu5859Isup2.hkl

Supporting information file. DOI: 10.1107/S2056989015013468/xu5859Isup3.cml

**Fig. 1.**
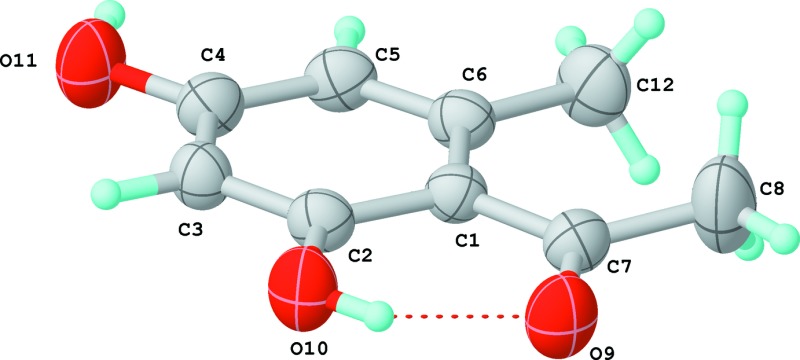
The molecular structure of the title compound with displacement ellipsoids drawn at the 50% probability level.

. DOI: 10.1107/S2056989015013468/xu5859fig1.tif
The mol­ecular structure of the title compound with displacement ellipsoids drawn at the 50% probability level.

**Fig. 2.**
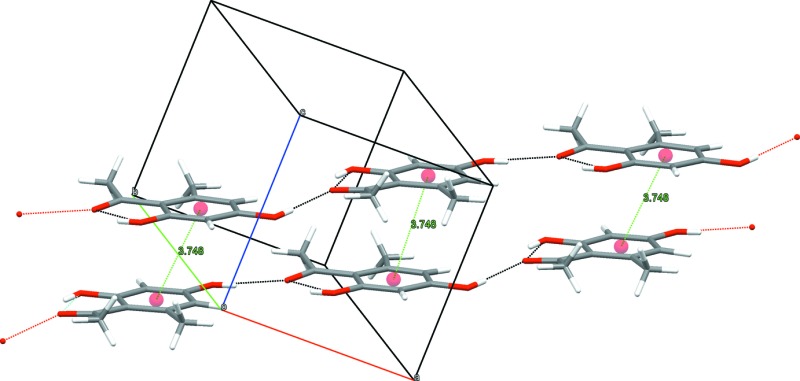
The partial packing diagram shows layers of molecules built up by bifurcated O—H···O hydrogen bonds and π–π intermolecular interactions between phenyl rings.

. DOI: 10.1107/S2056989015013468/xu5859fig2.tif
The partial packing diagram shows layers of mol­ecules built up by bifurcated O—H⋯O hydrogen bonds and π–π inter­molecular inter­actions between phenyl rings.

CCDC reference: 1412605

Additional supporting information:  crystallographic information; 3D view; checkCIF report

## Figures and Tables

**Table 1:** Hydrogen-bond geometry (, )

*D*H*A*	*D*H	H*A*	*D* *A*	*D*H*A*
O10H10O9	0.82	1.77	2.4991(16)	147
O11H11O9^i^	0.82	1.97	2.7843(16)	173

Symmetry code: (i) 
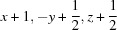
.

## supplementary crystallographic information

### S1. Comment

The title compound C_9_H_10_O_3_ (Fig. 1), 2,4-di­hydroxy-6-methyl­aceto­phenone [systematic name: 1-(2,4-di­hydroxy-6-methyl­phenyl) ethanone], was a penta­ketide secondary metabolite isolated from the culture media of the endophytic fungus *Nodulisporium* sp. Derivatives of this aceto­phenone have been demonstrated to possess inter­esting pharmacological activities, such as inhibition of lettuce seeds (Tabuchi *et al.*, 2014), bacterial plasmid transfer inhibition (Das and Khosla, 2009) and anti­cancer activity (Suzuki *et al.*, 2006). It is an important biosynthesis precursor for a large varieties of bioactive polyketides.

The geometric parameters of this compound (Fig. 2) are comparable with previously reported values of similar aceto­phenone compounds (Azeezaa *et al.*, 2009; Chakkaravarthi *et al.*, 2007a; Hill *et al.*, 2012). The bond lengths of C7—C8 and C6—C12 (1.4970 (2) and 1.5050 (2) Å), longer than that of C1—C7 (1.4580 (2) Å), may be a result of a resonant effect between C7—O9 (1.2485 (18) Å) carbonyl group and the aromatic ring. The bond length of C2—O10 (1.3466 (18) Å) is shorter than C4—O11 (1.3573 (19) Å). This may be a result of O10 being involved in an intra­molecular hydrogen bond. The acetyl group is coplanar with the aromatic ring C2—C1—C7—O9 (dihedral angle of 7.2 (2) °). The torsion angles C7—C1—C6—C12 and C7—C1—C6—C5 [1.9 (2)° and -179.75 (13)°, respectively] indicate a planar conformation of the respective moieties. An intra­molecular hydrogen bond was observed between the 2-phenolic hydrogen to the carbonyl group, O10—H10···O9 (D—H···A= 2.4983 (16) Å and O—H···O = 145°) to hold a carbonyl functionality in the coplanar plane of the aromatic ring. Inter­molecular hydrogen bonds between the 4-hydroxyl group to the carbonyl oxygen O11—H11···O9 (D—H···A= 2.7862 (18) Å and O—H···O = 171°) link the molecules in to an extended polymeric structure (Fig. 1). The π···π stacking inter­molecular inter­actions between two aromatic rings (centriod C1—C6) with a distance of (3.7478 (8) Å) (Fig. 2), further stabilize the three-dimensional network.

### S2. Experimental

The culture media of the endophytic fungus *Nodolisporium* sp. (10 L) were extracted with EtOAc (6 x 500 mL). After removal of the solvent under reduced pressure, the EtOAc extract (2.25 g) was subjected to column chromatography over silica gel eluting with EtOAc:hexane (30-100%), followed by MeOH:EtOAc (0-100%) to yield fractions 1-19. After combination and removal of the solvents, fraction 4 (162.9 mg) was further purified by Sephadex LH-20 (20% H_2_O-MeOH) to yield 2,4-di­hydroxy-6-methyl­aceto­phenone (133 mg). Single crystals were obtained by slow evaporation from EtOAc solution.

### S3. Refinement

The methyl H atoms were constrained to an ideal geometry with C—H distances of 0.98 Å and each group was allowed to rotate freely about its C—C bond. All other hydrogen atoms were placed in idealized locations (C—H = 0.96–0.98 Å, O—H = 0.82 Å) and refined as riding with U_iso_(H) = 1.2U_eq_(C) or 1.5U_eq_(O, methyl C).

### Crystal data

**Table:**

C_9_H_10_O_3_	*F*(000) = 352
*M**_r_* = 166.17	*D*_x_ = 1.367 Mg m^−^^3^
Monoclinic, *P*2_1_/*c*	Mo *K*α radiation, λ = 0.71073 Å
*a* = 7.3570 (3) Å	Cell parameters from 1754 reflections
*b* = 15.001 (1) Å	θ = 1.0–27.5°
*c* = 7.3180 (5) Å	µ = 0.10 mm^−^^1^
β = 91.017 (4)°	*T* = 298 K
*V* = 807.50 (8) Å^3^	Block, yellow
*Z* = 4	0.25 × 0.25 × 0.25 mm

### Data collection

**Table:**

Nonius KappaCCD diffractometer	1319 reflections with *I* > 2σ(*I*)
Radiation source: fine-focus sealed tube	*R*_int_ = 0.028
Detector resolution: 9 pixels mm^-1^	θ_max_ = 27.5°, θ_min_ = 3.1°
CCD scans	*h* = −9→9
3260 measured reflections	*k* = −17→19
1828 independent reflections	*l* = −9→9

### Refinement

**Table:**

Refinement on *F*^2^	Secondary atom site location: difference Fourier map
Least-squares matrix: full	Hydrogen site location: inferred from neighbouring sites
*R*[*F*^2^ > 2σ(*F*^2^)] = 0.053	H-atom parameters constrained
*wR*(*F*^2^) = 0.150	*w* = 1/[σ^2^(*F*_o_^2^) + (0.0739*P*)^2^ + 0.1187*P*] where *P* = (*F*_o_^2^ + 2*F*_c_^2^)/3
*S* = 1.03	(Δ/σ)_max_ < 0.001
1828 reflections	Δρ_max_ = 0.22 e Å^−^^3^
114 parameters	Δρ_min_ = −0.17 e Å^−^^3^
0 restraints	Extinction correction: *SHELXL2013* (Sheldrick, 2015), Fc^*^=kFc[1+0.001xFc^2^λ^3^/sin(2θ)]^-1/4^
Primary atom site location: structure-invariant direct methods	Extinction coefficient: 0.06 (2)

### Special details

**Table:**

Geometry. All e.s.d.'s(except the e.s.d. in the dihedral angle between two l.s. planes) are estimated using the full covariance matrix. The cell e.s.d.'s are taken into account individually in the estimation of e.s.d.'s in distances, angles and torsion angles; correlations between e.s.d.'s in cell parameters are only used when they are defined by crystal symmetry. An approximate (isotropic) treatment of cell e.s.d.'s is used for estimating e.s.d.'s involving l.s. planes.
Refinement. Refinement of F2 against ALL reflections. The weighted *R*-factor *wR* and goodness of fit *S* are based on F2, conventional *R*-factors *R* are based on *F*, with *F* set to zero for negative F2. The threshold expression of F2 > σ(F2) is used only for calculating *R*-factors(gt) *etc*. and is not relevant to the choice of reflections for refinement. *R*-factors based on F2 are statistically about twice as large as those based on *F*, and *R*-factors based on ALL data will be even larger.

### Fractional atomic coordinates and isotropic or equivalent isotropic displacement parameters (Å^2^)

**Table:**

	*x*	*y*	*z*	*U*_iso_*/*U*_eq_	
C1	0.70787 (19)	0.22482 (9)	0.77437 (17)	0.0385 (4)	
C2	0.70538 (18)	0.32006 (10)	0.77086 (18)	0.0407 (4)	
C3	0.8510 (2)	0.37042 (10)	0.83381 (19)	0.0436 (4)	
H3	0.8441	0.4381	0.8326	0.052*	
C4	1.00510 (19)	0.32826 (10)	0.89794 (19)	0.0447 (4)	
C5	1.0142 (2)	0.23520 (10)	0.9001 (2)	0.0446 (4)	
H5	1.1320	0.2021	0.9410	0.053*	
C6	0.87053 (19)	0.18331 (10)	0.84171 (17)	0.0416 (4)	
C7	0.5457 (2)	0.17763 (10)	0.70990 (19)	0.0456 (4)	
C8	0.5184 (3)	0.07921 (13)	0.7285 (3)	0.0776 (6)	
H8A	0.6044	0.0483	0.6544	0.116*	
H8B	0.3972	0.0640	0.6891	0.116*	
H8C	0.5360	0.0622	0.8541	0.116*	
O9	0.41657 (15)	0.21912 (7)	0.63688 (18)	0.0588 (4)	
O10	0.55972 (15)	0.36627 (7)	0.70969 (17)	0.0552 (4)	
H10	0.4830	0.3316	0.6688	0.083*	
O11	1.14661 (16)	0.37918 (8)	0.95736 (18)	0.0631 (4)	
H11	1.2256	0.3471	1.0026	0.095*	
C12	0.9014 (3)	0.08414 (11)	0.8510 (2)	0.0607 (5)	
H12A	0.8165	0.0580	0.9335	0.073*	
H12B	1.0231	0.0725	0.8940	0.073*	
H12C	0.8842	0.0588	0.7315	0.073*	

### Atomic displacement parameters (Å^2^)

**Table:**

	*U*^11^	*U*^22^	*U*^33^	*U*^12^	*U*^13^	*U*^23^
C1	0.0408 (8)	0.0373 (8)	0.0373 (7)	0.0021 (6)	−0.0033 (6)	0.0009 (5)
C2	0.0384 (7)	0.0409 (8)	0.0426 (7)	0.0061 (6)	−0.0036 (5)	0.0034 (6)
C3	0.0438 (8)	0.0373 (8)	0.0496 (8)	0.0011 (6)	−0.0048 (6)	0.0029 (6)
C4	0.0404 (8)	0.0485 (9)	0.0449 (8)	−0.0015 (6)	−0.0053 (6)	0.0013 (6)
C5	0.0402 (8)	0.0481 (9)	0.0452 (8)	0.0092 (6)	−0.0059 (6)	0.0017 (6)
C6	0.0464 (8)	0.0393 (8)	0.0390 (7)	0.0068 (6)	−0.0027 (6)	0.0024 (5)
C7	0.0462 (8)	0.0469 (9)	0.0436 (8)	−0.0020 (7)	−0.0047 (6)	0.0001 (6)
C8	0.0771 (13)	0.0495 (11)	0.1050 (15)	−0.0156 (9)	−0.0344 (11)	0.0089 (10)
O9	0.0443 (7)	0.0558 (7)	0.0756 (8)	−0.0015 (5)	−0.0172 (5)	0.0004 (5)
O10	0.0447 (7)	0.0420 (6)	0.0782 (8)	0.0077 (5)	−0.0177 (5)	0.0043 (5)
O11	0.0475 (7)	0.0556 (7)	0.0853 (9)	−0.0071 (5)	−0.0213 (6)	0.0027 (6)
C12	0.0671 (11)	0.0429 (9)	0.0717 (11)	0.0126 (8)	−0.0123 (8)	0.0029 (8)

### Geometric parameters (Å, º)

**Table:**

C1—C6	1.4288 (18)	C6—C12	1.506 (2)
C1—C2	1.429 (2)	C7—O9	1.2481 (18)
C1—C7	1.4585 (19)	C7—C8	1.497 (2)
C2—O10	1.3462 (16)	C8—H8A	0.9600
C2—C3	1.3831 (19)	C8—H8B	0.9600
C3—C4	1.374 (2)	C8—H8C	0.9600
C3—H3	1.0170	O10—H10	0.8200
C4—O11	1.3566 (18)	O11—H11	0.8200
C4—C5	1.398 (2)	C12—H12A	0.9600
C5—C6	1.375 (2)	C12—H12B	0.9600
C5—H5	1.0380	C12—H12C	0.9600
			
C6—C1—C2	116.89 (13)	O9—C7—C1	120.57 (14)
C6—C1—C7	125.12 (13)	O9—C7—C8	115.38 (14)
C2—C1—C7	117.99 (12)	C1—C7—C8	124.04 (14)
O10—C2—C3	115.89 (13)	C7—C8—H8A	109.5
O10—C2—C1	122.05 (13)	C7—C8—H8B	109.5
C3—C2—C1	122.04 (12)	H8A—C8—H8B	109.5
C4—C3—C2	119.47 (14)	C7—C8—H8C	109.5
C4—C3—H3	120.3	H8A—C8—H8C	109.5
C2—C3—H3	120.3	H8B—C8—H8C	109.5
O11—C4—C3	118.31 (14)	C2—O10—H10	109.5
O11—C4—C5	121.49 (13)	C4—O11—H11	109.5
C3—C4—C5	120.19 (13)	C6—C12—H12A	109.5
C6—C5—C4	121.71 (13)	C6—C12—H12B	109.5
C6—C5—H5	116.9	H12A—C12—H12B	109.5
C4—C5—H5	121.4	C6—C12—H12C	109.5
C5—C6—C1	119.68 (13)	H12A—C12—H12C	109.5
C5—C6—C12	115.52 (13)	H12B—C12—H12C	109.5
C1—C6—C12	124.79 (14)		
			
C6—C1—C2—O10	179.79 (12)	C4—C5—C6—C1	0.9 (2)
C7—C1—C2—O10	−0.3 (2)	C4—C5—C6—C12	179.57 (14)
C6—C1—C2—C3	−1.66 (19)	C2—C1—C6—C5	0.41 (19)
C7—C1—C2—C3	178.24 (13)	C7—C1—C6—C5	−179.48 (13)
O10—C2—C3—C4	−179.77 (13)	C2—C1—C6—C12	−178.14 (14)
C1—C2—C3—C4	1.6 (2)	C7—C1—C6—C12	2.0 (2)
C2—C3—C4—O11	179.41 (13)	C6—C1—C7—O9	−172.96 (13)
C2—C3—C4—C5	−0.2 (2)	C2—C1—C7—O9	7.2 (2)
O11—C4—C5—C6	179.36 (14)	C6—C1—C7—C8	8.5 (2)
C3—C4—C5—C6	−1.0 (2)	C2—C1—C7—C8	−171.41 (16)

### Hydrogen-bond geometry (Å, º)

**Table:**

*D*—H···*A*	*D*—H	H···*A*	*D*···*A*	*D*—H···*A*
O10—H10···O9	0.82	1.77	2.4991 (16)	147
O11—H11···O9^i^	0.82	1.97	2.7843 (16)	173

Symmetry code: (i) *x*+1, −*y*+1/2, *z*+1/2.
