# Supplementary figures and images for: Crystal structure of 1-(2,4-dihy­droxy-6-methyl­phen­yl)ethanone
Source: Acta Crystallogr E Crystallogr Commun. 2015 Jul 29;71(Pt 8):o612–3. doi: 10.1107/S2056989015013468 (PMC4571424; doi:10.1107/S2056989015013468)

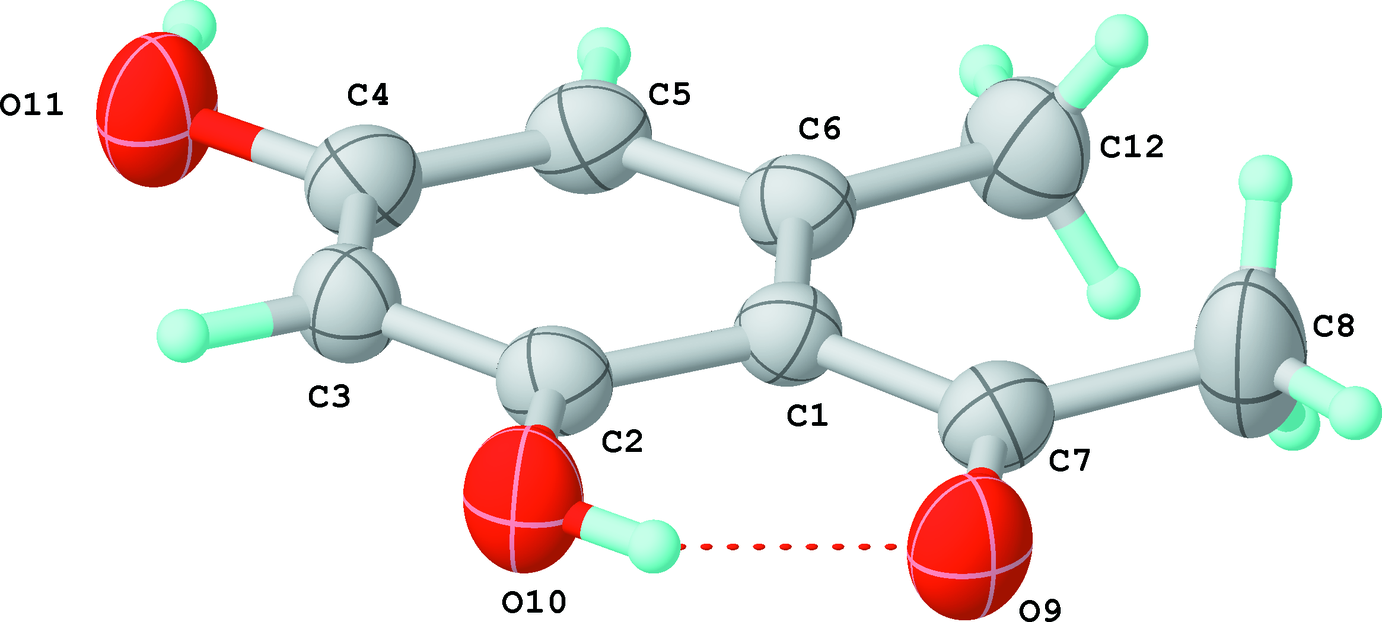

Supplement: Supplementary file 4 [file e-71-0o612-fig1.tif]

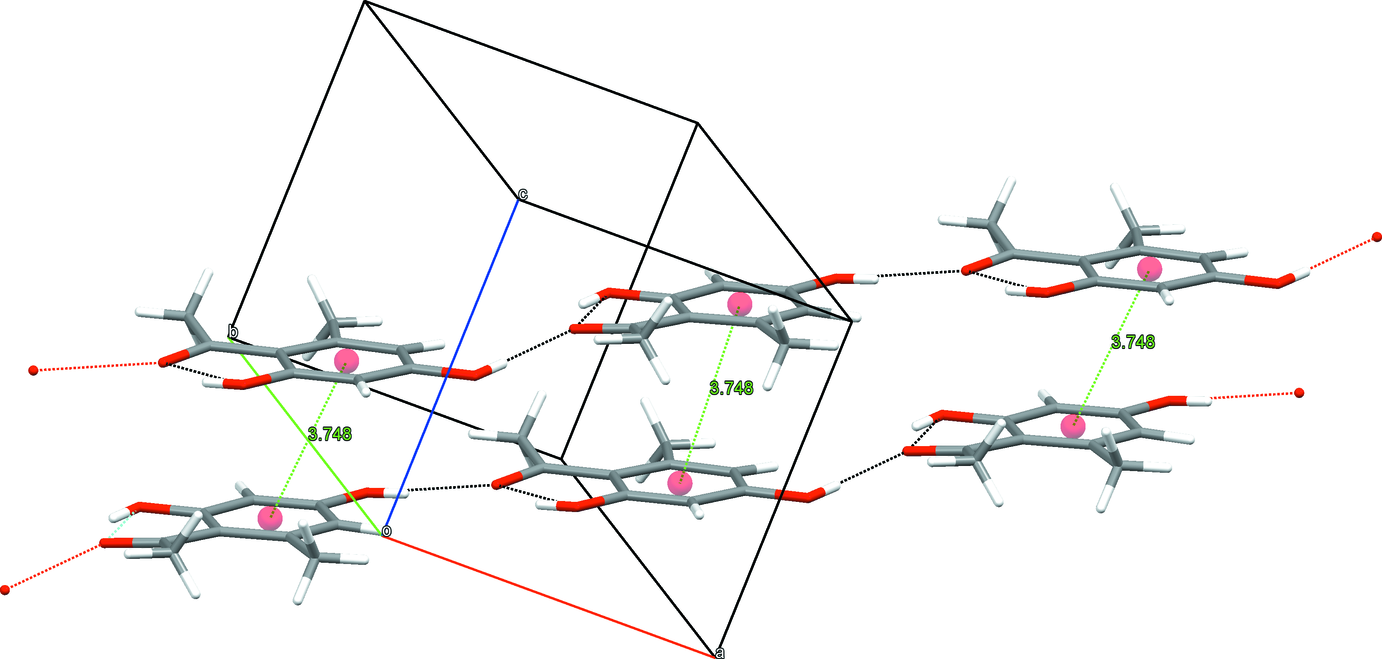

Supplement: Supplementary file 5 [file e-71-0o612-fig2.tif]
